# Long-Term Monitoring of Individuals With Chronic Obstructive Pulmonary Disease Using Digital Health Technology: Qualitative Study

**DOI:** 10.2196/63660

**Published:** 2025-02-05

**Authors:** Shih-Ying Chien, Han-Chung Hu, Hsiu-Ying Cho

**Affiliations:** 1 Department of Industrial Design Chang Gung University Taoyuan Taiwan; 2 Department of Public Health & Medical Humanities School of Medicine National Yang Ming Chiao Tung University Hsinchu Taiwan; 3 Department of Physical Medicine and Rehabilitation Linkou Chang Gung Memorial Hospital (CGMH) Taoyuan Taiwan; 4 Department of Thoracic Medicine Linkou Chang Gung Memorial Hospital Taoyuan Taiwan; 5 Department of Respiratory Therapy Linkou Chang Gung Memorial Hospital Taoyuan Taiwan

**Keywords:** digital health, chronic obstructive pulmonary disease, digital health technology, barriers and facilitators, Taiwan

## Abstract

**Background:**

Digital health adoption in clinical practice has been widespread, yet there remains further potential for optimizing care specifically for chronic obstructive pulmonary disease (COPD). This study therefore conducted qualitative research involving 35 health care professionals from a range of hospitals in Taiwan.

**Objective:**

This study aims to investigate barriers and facilitators related to the implementation of digital health technology (DHT) in the long-term monitoring of individuals with COPD based on clinical experiences in Taiwan. The perspectives of Taiwanese health care professionals provided valuable insights into the challenges and opportunities associated with using DHT for the management and enhancement of respiratory rehabilitation and long-term monitoring of patients with COPD.

**Methods:**

Several key themes related to the development of DHT were identified. Barriers encompassed concerns pertaining to digital safety, insurance coverage, constraints related to medical resources, and the presence of a digital divide. Facilitators included the potential for cost reduction, personalized prescriptions, and instilling motivation in users.

**Results:**

To enhance the acceptance and use of DHT, embracing a user-centered approach that prioritizes the distinct needs of all parties involved is recommended. Moreover, optimizing and leveraging the effective use of DHT in managing the health of individuals with COPD promises to deliver care characterized by greater precision and efficiency.

**Conclusions:**

Overall, the benefits of using DHT for the long-term care of patients with COPD outweigh the disadvantages. After the COVID-19 pandemic, there has been an increased emphasis in Taiwan on the effectiveness of DHT in managing chronic diseases. Relevant studies including this paper have suggested that web-based exercise management systems could benefit patients with COPD in rehabilitation and tracking. Our findings provide meaningful directions for future research endeavors and practical implementation. By addressing identified barriers and capitalizing on facilitators, advancements can be made in the development and use of DHT, especially in overcoming challenges such as information security and operational methods. The implementation of the recommended strategies will likely lead to improved COPD care outcomes.

## Introduction

Chronic obstructive pulmonary disease (COPD) is a major public health problem and is the third-leading cause of morbidity and mortality by disease worldwide [1,2]. Indeed, over 328 million people were diagnosed with this disease in 2019 [2,3]. Caused by smoking and air pollution [4,5], COPD is typically associated with reduced physical, emotional, and social functioning, resulting in a decline in overall quality of life [4,5]. Health care professionals have reported that physical activity and effective self-management are strongly correlated with improved lung function [6,7], reduced exacerbations, and significant improvements in the quality of life for hospitalized and discharged patients [7,8]. However, decreased exercise tolerance is linked with dyspnea, which is the primary symptom experienced by individuals with COPD [9-11]. Consequently, this symptom often leads to poor adherence to self-care management and exercise in patients with COPD [12]. Research has shown that lower levels of physical activity are associated not only with increased hospitalization but also with an elevated risk of premature death [13,14]. Thus, respiratory rehabilitation plays a vital role in improving lung function and aiding recovery in patients with lung damage or injury [15].

To enhance adherence to exercise and facilitate health management in patients with COPD, the implementation of digital health (DH) has been suggested as an innovative approach [16,17]. DH models offer opportunities to optimize COPD care by leveraging accumulated big data to assess individual exercise capacity and provide personalized exercise prescriptions, thereby enhancing treatment efficacy [18]. DH primarily involves the application of information and communications technologies, leading to the emergence of concepts such as mHealth or eHealth [19,20]. Digital health technology (DHT) refers to technologies enabling the remote monitoring of physiological markers such as oxygen saturation and the self-monitoring of physical activity using wearable activity trackers. It also encompasses the use of web-based apps to support disease management, facilitate behavioral change, provide medication reminders, and predict personalized exercise prescriptions.

Compared to alternative therapies, digital pulmonary rehabilitation is a cost-effective and beneficial intervention that encompasses various components, including diagnosis, training, and evaluation [21-23]. This comprehensive approach effectively delays the deterioration of lung function and slows the progression of the disease [24,25]. Moreover, digital pulmonary rehabilitation empowers patients to have real-time control over their condition [26,27]. Furthermore, it benefits health care professionals by enabling the collection of longitudinal health data from patients, thereby facilitating informed decision-making and the provision of personalized care [28,29].

However, systematic reviews have found no significant evidence demonstrating the effective impact of digital pulmonary rehabilitation on COPD management [30,31]. This could be attributed to the advanced age or limited motivation for exercise in the majority of patients with COPD, who often have impaired lung function. Additionally, their limited familiarity with digital spaces, devices, and knowledge further contributes to this issue [32,33]. Notably, prior exploration of the needs and preferences of health care professionals and patients as well as careful consideration of intervention design are essential factors that have rarely been addressed in the implementation of digital pulmonary rehabilitation for patients with COPD [34,35]. Failure to consider the needs of health care professionals and patients from a user-centric perspective may lead to user experience issues, such as lack of clinical use, disruption of patients’ workflow, and negative engagement experiences with DHT [36,37]. Consequently, users may become unwilling to continue using and adhering to treatment regimens [38-40].

Prior to conducting the primary survey in this study, we conducted informal interviews with health care professionals within the hospital to gather their perspectives on using DHT for managing remote pulmonary rehabilitation. Although the sample size was small (n=10), the majority of health care professionals emphasized that remote rehabilitation is an important development for the future. This was particularly evident during the COVID-19 outbreak when many hospitals faced closures, preventing patients from continuing their rehabilitation programs and hindering their overall recovery progress. Most Health care professionals believed that implementing DH models for respiratory rehabilitation was necessary. However, both health care professionals and patients had limited knowledge about DH. Moreover, despite the advancements in medical technology in Taiwan, the application of DH in the care of patients with COPD remains subject to various barriers, such as information security measures, disparities between urban and rural areas, and the acceptance of digital tools among professional caregivers.

This study explores a broader range of barriers to using DHT in respiratory rehabilitation from the perspective of health care professionals. We also assessed the feasibility of implementing DHT in the management of COPD. Given Taiwan’s relatively rich medical resources and its urban-rural disparities, the findings from this research serve as a valuable reference for similar implementations worldwide. This study not only contributes to optimizing clinical rehabilitation designs of DH from a user perspective but also significantly enhances the benefits for patients throughout the treatment process.

## Methods

### Study Design

A qualitative descriptive methodology was used to investigate the barriers encountered when using DH for managing pulmonary rehabilitation among patients with COPD. A snowball sampling technique was used to recruit 35 diverse health care professionals in this study. The participants included 12 respiratory physiotherapists, 8 respiratory specialist nurses, 5 rehabilitation physicians, 2 thoracic surgeons, 5 physical therapists, and 3 sports medicine specialists. The interviewees were representatives in the field of cardiopulmonary rehabilitation in Taiwan, with an average age of 52 (SD 8) years and a minimum of 6.3 (SD 7.4) years of relevant work experience.

### Ethical Considerations

To investigate the key elements of pulmonary rehabilitation for patients across different regions, this study was conducted in the Department of Physical Medicine and Rehabilitation across 6 affiliated hospitals situated in different regions of Taiwan. The research protocol received approval from the Research Ethics Board of Chang Gung Hospital (202200070B0). All participants possessed experience in providing care for patients with COPD. Before the commencement of interviews, participants were provided with physical copies of consent documents. All individuals who participated in the study provided informed written consent. Among these consents, 1 copy was retained by the researcher, while another was retained by the participant. Subsequently, semistructured one-on-one telephone interviews were conducted, each lasting approximately 45 to 60 minutes. The interview data were processed with unique identifiers to ensure participants’ rights. All participants voluntarily participated in the study without receiving any compensation, ensuring the fairness of the experiment.

### Procedure

The study was conducted in June 2022 and completed in May 2023. All interviews were conducted by the first author (SYC), who is an experienced qualitative researcher. The interviews were carried out in a one-to-one semistructured format. An interview topic guide in Textbox 1, outlined in Tables 1 and 2, was used as a framework for the interviews. The initial version of the topic guide was developed based on relevant literature and consultations with respiratory therapists and rehabilitation physicians. The interviews were designed to gather information through semistructured and open-ended questions, allowing for in-depth discussions on the interview content.

**Interview topic guide (including clinical, home care, and digital health aspects).**

**Clinical perspective**

- Please outline the basic requirements for conducting pulmonary exercise training for patients with chronic obstructive pulmonary disease (COPD).
- Please describe the information and physiological parameters that need to be collected for conducting pulmonary exercise training.
- Please describe the current limitations and challenges in clinical respiratory care.

**Self-care management**

- Please describe health management and follow-up methods for inpatients and discharged patients.
- Please discuss the key factors influencing patients' self-management abilities.
- Please describe the difficulties of health management for patients after discharge.

**Digital health**

- Please describe your views on digital health (DH).
- Please describe the benefits and challenges that DH may bring to patients with COPD.
- Please describe your experiences of implementing DH in clinical practice. What are your views on incorporating DH into respiratory rehabilitation?
- Please describe the factors that need to be considered when integrating DH into the workflow of respiratory rehabilitation care.
- Please describe the potential risks of using DH technology in clinical settings.
- Please describe the indicators that clinicians wish to track for patients after discharge.

**Table 1:** Participant demographics and characteristics (N=35).

	Values
Sex (male:female), n:n	18:17
Age (years), range (mean, SD)	37-67 (52, 8)
Years of experience in pulmonary rehabilitation, range (mean, SD)	5-36 (6.3, 7.4)

**Table 2:** Health care professionals in pulmonary rehabilitation.

Type of health care professionals	Participants, n (%)
Respiratory physiotherapists	12 (34)
Respiratory specialist nurses	8 (23)
Rehabilitation physicians	5 (14)
Thoracic surgeons	2 (6)
Physical therapists	5 (14)
Sports medicine specialists	3 (9)

### Data Analysis

Participants were informed that all study data would be treated anonymously. Each participant voluntarily consented to join the study, with no compensation offered during the research period. The entire interview process was audio-recorded, and unique identifiers were assigned to each participant to ensure confidentiality. The transcripts were generated based on the interview topic guide, and NVivo software (version 12.2; QSR International) was used to conduct a thematic analysis (TA) of the transcripts.

This study follows the reflexive TA method developed by Braun and Clarke [41,42], using a coding-first approach to identify themes that help understand and construct the clinical experiences of health care providers. This approach aims to authentically capture the participants’ perspectives on events and uncover the meanings behind surface-level expressions. The TA consists of 6 meticulous steps, ensuring consistency, logic, and comprehensiveness in the research process. It has been demonstrated to possess confirmability, reliability, and credibility [41,42]. Moreover, this descriptive method distinctly articulates the researcher’s interpretation of the data, rather than simply reiterating the participants’ statements.

The analysis of the interviews followed an inductive and cyclical process. This involved a thorough reading and rereading of each transcript to gain familiarity with the data [43]. The data were then coded with unique labels, and themes and subthemes were generated based on the coded data. To ensure analytical rigor, the first author independently coded the data. Then, 2 experienced researchers were invited to review and validate the coding process.

In reference to the study by Hennink and Kaiser [44] on sample sizes for saturation in qualitative research, this research also considers the work of Rowlands et al [45], which proposes calculating thematic saturation using a lognormal distribution with a selected confidence level. In this study, the data obtained from 35 participants is expected to hold significant reference value, aligning with these methodological guidelines [44,45].

## Results

### Overview

After nearly a year of duration, this study completed interviews with 35 participants. Based on the interview records, an analysis was conducted, resulting in the generation of themes and subthemes for this study. The analysis has identified several themes: digital safety and reliability, insurance coverage and safety concerns, medical resource constraints, and the digital divide, which are presented as barriers. Additionally, the themes of the advantages of using DHT, reduced health care expenditure, personalized prescribing, and motivation for use are presented as facilitators.

### Barriers

#### Digital Safety and Reliability

The majority of the interviewees (n=21, 60%) expressed concerns about the use of DHT in health care services, particularly in relation to information security and patient privacy. As the primary objective of health care is to ensure patient safety, any potential breach of privacy resulting from seeking medical treatment is considered harmful to patients. Therefore, information security in the health care field is a highly sensitive issue for patients. The promotion of DHT in the health care industry is critically dependent on network security and data protection.

Furthermore, more than half of the interviewees (n=19, 54%) had concerns about the accuracy of the generated data. Inaccurate data have the potential to lead to erroneous medical decisions and in severe cases to even jeopardize the health and safety of patients.

Patient information is highly sensitive, and any data breaches or information errors in a hospital can result in significant losses. While I acknowledge the many benefits that DHT can bring, I believe that ensuring information security and data accuracy should be a minimum requirement.

Thoracic surgeon 26

Technology has been advancing rapidly, especially after the pandemic. Currently, almost every industry relies on the internet to handle various aspects of daily life. However, the healthcare field is different. There are two main concerns. First, there is a fear of data breaches and the potential leakage of sensitive information. Second, there is a worry that if the machines make mistakes, it could jeopardize the safety and well-being of patients.

Respiratory physiotherapist 3

Regardless of everything, patient safety always comes first for healthcare professionals. Digital health is just starting to thrive, and it seems to bring much convenience. However, I believe there is still room for improvement. For example, how do you prove that it is accurate?

Thoracic surgeon 27

The interviewees highlighted the issue of low patient adherence to medical instructions, particularly among patients with COPD, which encompasses various complex aspects such as medication adherence, exercise control, and smoking cessation. Most interviewees emphasized that patients with COPD tend to dislike exercise due to the physical burden it imposes (n=26, 74%), as they believe it may cause discomfort and even pose a threat to their livelihood. Consequently, after being discharged from the hospital, patients often struggle to comply with basic exercise instructions, resulting in a decline in pulmonary function. Even with the use of optimal rehabilitation assistive devices, there remains an insufficient improvement in the pulmonary function of patients. Moreover, the interviewees (n=19, 54%) expressed concerns regarding the long-term observation and accumulation of training hours required to assess patients’ recovery progress in pulmonary function training. Consequently, when using DHT, there may be concerns about the reliability of generated medical recommendations if the accumulated data are incomplete or if the duration of data collection is insufficient to provide meaningful results.

Patients can be categorized into inpatients and outpatients. Generally, inpatients tend to be more compliant and follow the doctor's treatment instructions. Managing the health of outpatients can be challenging because many patients become difficult to reach once they return home, and it is uncertain whether they are taking medication and engaging in physical activity as prescribed.

Respiratory physiotherapist 1

Our patients are not very keen on physical activity because it often triggers breathlessness and discomfort. This makes it challenging for them to engage in exercise. Many patients who cooperate well during their hospital stay exhibit different behaviors once they return home. Therefore, for patients with chronic conditions such as COPD, it is crucial that we have digital health technology to assist us in monitoring their condition closely.

Respiratory physiotherapist 9

After being discharged, patients often resume smoking, engage in unhealthy eating habits, and neglect physical activity due to a lack of supervision. As a result, their lung function deteriorates rapidly, leading to a cycle of readmissions until their unfortunate demise.

Respiratory physiotherapist 6

After discharge, patients often lack motivation and health awareness, which lead to poor compliance with medical instructions. Some may occasionally follow them, while others resist any form of supervision. Both types of patients are not suitable candidates for digital health technology-based healthcare prescription advice because insufficient information would be collected, which may result in inaccurate prescriptions and potential harm to their health.

Respiratory specialist nurse 17

#### Insurance Coverage and Safety Concerns

Prior to the outbreak of the COVID-19 pandemic, global insurance coverage for telemedicine was subject to stringent limitations. However, as the severity of the pandemic increased, regions including Taiwan gradually relaxed regulations surrounding telehealth. Nevertheless, participants universally emphasized that DHT continues to face multiple constraints and considerations. Specifically, not all medical conditions are suitable for DHT adoption, and a lack of sufficient information impedes health care professionals from making accurate medical decisions. Moreover, vulnerable patients, particularly elderly patients, often face potential risks of falls and emergencies.

In the past, telemedicine was not very successful because insurance coverage was limited. Even though there are more flexible rules now due to the pandemic, digital health still has its challenges. Some diseases cannot be diagnosed through video calls alone, and there are also concerns about risks for weaker patients, such as the possibility of accidents or falls.

Rehabilitation physician 22

I believe digital health technology can play a significant role in managing health information, while the diagnosis and treatment depend on the situation. Of course, conditions vary in different regions, so I think digital health technology should consider factors such as disease type, severity, and the availability of alternative options in diagnosis and treatment.

Thoracic surgeon 26

#### Medical Resource Constraints

The interviewees discussed the challenges of implementing DHT in different hospitals, which stem from variations in health care resources. For urban medical centers, the promotion of DHT is relatively easier compared to regional hospitals located in remote rural or island areas. This is due to the limitations in health care resources, knowledge gaps, and a shortage of health care professionals in rural areas, leading to an increasing disparity in health care between urban and rural regions. However, it is precisely because of the scarcity of health care resources in remote rural and island areas that the introduction of DHT is needed in order to bridge the gap in health care access between urban and rural areas.

In my opinion, not every region can implement digital health technology because conditions vary. For example, in rural areas, many places lack internet signal, making it impossible for activities such as video consultations or data transmission to take place.

Sports medicine specialist 34

Urban and rural areas have different living conditions. In rural areas, apart from limited healthcare resources, there is also a shortage of staff. Additionally, there are disparities in education and professional expertise across different regions. As a result, the acceptance and trust in digital health technology vary among healthcare professionals and patients in different areas.

Physical therapist 31

Digital health technology can be a valuable tool for rural or island areas, but before considering its use in healthcare services, it is important to assess whether digital health technology is suitable for the specific region. This involves examining what technologies can address the local challenges, improve specific issues, and enhance overall benefits.

Respiratory specialist nurse 14

#### Digital Divide

As mentioned earlier, both health care professionals and patients may face digital disparities. These disparities are more prevalent in rural or island areas due to factors such as economic conditions, educational levels, living environments, and comorbidities, including cognitive impairments, which can potentially impact the opportunities and abilities of individuals to use digital products such as computers or the internet. Consequently, there exists a significant gap in the health care capacity for medical examinations, monitoring, and care between urban and rural areas.

Typically, COPD patients do not just have a single condition. They may also experience physical, cognitive, or visual impairments. This means they might face difficulties with tasks such as connecting to or operating computers, or expressing themselves.

Rehabilitation physician 21

“Based on my past experience, although we use smartphones daily for information, older doctors like us may find it challenging to practice and become proficient with new IT products, let alone the patients.

Thoracic surgeon 27

### Facilitators

#### Advantages of Using DHT

Interviewees emphasized the need for an adaptation period when adopting DHT (n=26, 74%), for both health care professionals and patients. They unanimously acknowledged the significant clinical benefits of using DHT to assist patients with COPD in respiratory rehabilitation. Moreover, they recognized the advantages of DHT in facilitating the recovery of emergency inpatients and monitoring discharged patients. For the majority of patients who are unable to remain hospitalized for an extended period due to insurance regulations, continuous monitoring and remote care treatment at home are essential. DHT is regarded as the optimal clinical tool choice in such cases.

I believe that every disease requires going through four stages: testing, treatment, monitoring, and health management. It is just a matter of the extent to which each stage is needed. COPD patients, in particular, require them because their condition does not improve, so having long-term tracking and health management tools is crucial to assist us in our clinical work.

Rehabilitation physician 22

Due to insurance regulations, most hospitalized patients are discharged within a certain number of days. If there are still relevant technologies or tools available for continuous remote monitoring or care of COPD patients after discharge, it would be a significant advancement.

Respiratory specialist nurse 14

#### Reduced Health Care Expenditure

Most interviewees noted that patients with COPD often face limitations in adhering to medical instructions (n=23, 66%), for example, taking medications and following exercise prescriptions, due to factors such as home environment, declining physical function, and transportation difficulties after discharge. The application of DHT is seen as an effective solution to mitigate these limitations.

Some patients face challenges in contacting or returning for follow-up care after discharge due to their residential or transportation constraints. If there is technology available to assist us in regularly monitoring discharged patients, their willingness to cooperate would likely be higher.

Respiratory specialist nurse 20

#### Personalized Prescriptions

Most participants highlighted the potential of DHT in enabling personalized prescriptions through the accumulation of data (n=24, 69%). They emphasized that the use of big data and machine learning can facilitate data processing, enabling the identification, inference, and prediction of individuals’ health statuses and required rehabilitation interventions based on changes in their health behaviors.

To be honest, I have high expectations for big data because humans cannot remember everything, but machines can. Moreover, the larger the data are, the more accurate their judgments become. It is a great clinical tool.

Thoracic surgeon 26

I believe we should understand that artificial intelligence does not provide a definitive answer, but it is certainly the best supporting evidence for making judgments.

Sports medicine specialist 35

#### Increased Motivation to Participate in Rehabilitation Programs

The motivation of patients to use DHT will be a key determinant of the success of its implementation. Our participants discussed how issues related to hardware and software devices, technology, and operation are likely to affect user motivation. While they generally recognized the convenience and clinical benefits of DHT, many pointed out the difficulties encountered by older health care professionals when learning new technology. Thus, the interface design of DHT must be optimized for users, both patient users and health care professionals. This will increase motivation to participate in remote rehabilitation programs.

Learning and adapting to new tools is not easy for us or for patients, so interface design is also a crucial factor.

Thoracic surgeon 27

I believe motivation plays a significant role in many things. If digital healthcare can help alleviate our clinical workload, of course, we are all willing to use it, as are the patients. If they know it is for their own good, I believe they will be more than willing to give it a try.

Rehabilitation physician 23

## Discussion

### Overview

This study provides insights into the perspectives of health care professionals on the use of DHT in the care of patients with COPD and explores potential issues faced by both the clinical and patient sides. The findings highlight the urgent need to promote DHT at the clinical level, particularly in the postpandemic era, to reduce interpersonal contact, interaction, and health care costs for hospitals and patients.

### Findings

Previous research has often neglected the user-centered perspectives and diverse needs of stakeholders when investigating DHTs. Additionally, the demands and feasibility of DHT vary across regions due to population demographics and contextual differences. Addressing these challenges requires tailored investigations of stakeholder needs, encompassing health care providers and users, considering specific regional characteristics. Collaborative or participatory design approaches can facilitate the development of innovative DHT solutions that are adaptable to different regional contexts, including urban, rural, and island areas, with the ultimate goal of maximizing the benefits of DHTs in health care.

In summary, this study reveals that due to significant individual differences among patients with COPD, a singular treatment approach cannot be universally applicable, and a uniform health care service model cannot be implemented across regions. Respondents preferred personalized prescriptions that consider crucial factors such as patients’ health conditions, geographic locations, and educational levels, thus facilitating the provision of diverse DH services.

Furthermore, the aging population and lifestyle changes in the postpandemic era have underscored the importance of pulmonary rehabilitation as a key indicator for DHT development. The findings indicate that both health care professionals and patients have unmet digital literacy needs, emphasizing the importance of considering usability when implementing DHT and constructing pulmonary rehabilitation systems. This study contributes valuable insights into the demands and preferences of health care professionals regarding DHT in the care of patients with COPD. The user-centric design approach not only benefits health care professionals in clinical care for inpatients but also expands the applications of DHT in postdischarge monitoring, observation, and ongoing health care services for patients with COPD.

Despite health care professionals having concerns about the health information obtained from patients using DHT, they still recognize numerous advantages of DHT in clinical practice. For instance, DHT can streamline repetitive clinical tasks and proactively collect patient data through recommended system and device designs, thereby reducing the chances of human errors in documentation and medical decision-making. Simultaneously, simplified hardware and software designs can facilitate the collection and management of patient exercise levels and fundamental physiological data, particularly for discharged patients. Despite the mentioned information security issues, DHT faces additional risks and limitations in clinical applications.

Health care professionals acknowledge that insurance coverage and personal safety have hindered the successful implementation of DHT for many years [19]. Prior to the pandemic, many doctors faced challenges in understanding and navigating the insurance system, resulting in unsuccessful insurance claims. Furthermore, older patients may struggle with using internet-connected devices, making remote consultations difficult. Health care professionals also consider the advanced age of patients with COPD, raising concerns about potential falls during simple rehabilitation exercises at home. Additionally, various constraints in manpower, equipment, and infrastructure within the clinical setting hinder the widespread adoption of DHT. For instance, patients with severe respiratory disease rely on large respiratory devices and specialized personnel for treatment.

Past studies have identified resource constraints as the primary hindrance to the widespread adoption of DH, mainly due to the inability to generate reliable data. However, this investigation reveals that the lack of consideration for local geographical conditions and population structures has impeded the proliferation of DH. This results in a disconnect between health care providers and patients, either due to communication barriers or disparities in digital literacy, thereby reducing acceptance. It is important to acknowledge that different regions, including urban, rural, and island areas, exhibit varying living environments, necessitating the use of different DH tools to ensure the generation of precise, reliable, and trustworthy data. Moreover, given the significant individual differences among patients with COPD, a one-size-fits-all approach is deemed inappropriate. Consequently, emphasizing the significance of personalized prescriptions, this study highlights the potential of big data in achieving tailored and accurate treatments. Finally, contrary to previous research attributing patients’ inadequate self-management to a lack of motivation, this study proposes that their inability to effectively engage in health management may stem from practical constraints such as the unavailability of necessary equipment or the complexity of operations. In summary, by adopting a human-centered approach and considering the needs and circumstances of various stakeholders, the development of health care services can be optimized, ultimately enhancing patient adherence to medical instructions.

### Future Directions

The survey findings of this study indicate that the majority of health care professionals perceive the potential of DHT to facilitate the care of patients with COPD. However, both health care professionals and patients require a period of time to adapt to and become familiar with the use of digital tools. The novelty of this study lies in proposing that each disease undergoes 4 stages: detection, treatment, monitoring, and health management. However, the distribution of needs across these stages varies for different diseases. For instance, in the context of COPD, a condition that cannot be clinically cured, patients require long-term monitoring of their condition and self-health management. Therefore, DHT should be designed with individualized approaches for different diseases. Participants widely believed that DHT tools not only assist patients in understanding their own condition but also alleviate the burden of clinical work.

Previous studies have frequently cited inadequate compliance with exercise programs due to a lack of motivation and home environment factors. However, this study posits that the appropriate use of DHT can enhance patient motivation for exercise and reduce health care expenditures. By leveraging DHT, it becomes possible to synchronously execute consultations, treatments, monitoring, and overall health management for patients with COPD.

While the reliability of big data may still be questioned in many studies, the accumulation and learning capabilities of big data over the years will serve as a reference for future health care professionals when making medical decisions, given the inherent limitations of human memory and abilities. The information provided by DHT may not be the sole definitive answer, but it is certainly the best evidence supported by a scientific basis. The personalized prescriptions inferred by DHT are accumulated from individual health behavior changes, enabling the identification, inference, and prediction of individual needs for rehabilitative interventions. This will lead to more accurate health care decisions and provide an additional level of patient safety.

Finally, the issue of low patient adherence to health behaviors is often discussed in many studies. However, this problem also applies to health care professionals. Learning new things is inherently challenging for everyone, especially for older individuals. Therefore, for both patients and health care professionals, the motivation to use and accept DHT is crucial. If the use of DHT can help health care professionals reduce their clinical workload, make more accurate judgments, and lower the rate of medical decision errors, it is expected to increase the chances of clinical acceptance of DHT interventions. On the other hand, if DHT can assist patients in effectively managing and controlling their own health [20] while making the treatment process more interesting and reducing discomfort, it is believed that the majority of patients would be willing to try it. Of course, it is crucial that the devices, systems, or equipment designed for DHT adhere to design principles that are simple and intuitive and minimize errors. With this in mind, popularizing DHT should not be a challenging task.

### Study Limitations

Given the global attention to patients with COPD and the increasing demand for care and management of chronic lung diseases, along with the reliance on DHT during the pandemic, particularly in the health care sector, it has been confirmed that DHT can effectively perform the 4 major clinical tasks of diagnosis, treatment, monitoring, and health management even in special circumstances. Despite the rigorous investigation conducted over the course of one year, this study still has some limitations. First, the study used a snowball sampling method, which may introduce information bias and deviation due to the potential similarity of perspectives. Second, the study overlooked the age and work experience differences among health care professionals involved in the care of patients with COPD, resulting in a broad and unfocused information scope. Finally, the research solely relied on interview-based data collection without conducting a questionnaire survey, potentially leading to unverified differences between the interview findings and questionnaire responses.

Based on the above, this study represents a novel postpandemic reevaluation of the clinical applicability of DHT for managing patients with COPD. It aims to explore the potential of using DHT in enhancing health management for patients with COPD while considering the associated obstacles and potential benefits from a clinical perspective. According to the findings of the survey, the majority of health care professionals express concerns regarding the security and reliability of data generated by DHTs. These concerns can be attributed to two primary reasons: (1) apprehension regarding potential privacy breaches for patients; and (2) the lack of a validation platform for DHT, which raises doubts about its accuracy. Many respondents also mentioned incomplete data and potential risks to patient safety due to information security issues. Despite having high expectations for DHT, there is still perceived room for improvement. Existing research suggests poor adherence among patients with chronic diseases, especially patients with COPD who may exhibit reluctance to regular physical activity due to compromised lung function and struggle to maintain complete smoking cessation due to self-discipline challenges. Moreover, they face difficulties related to the absence of effective tracking and management systems. These factors hinder patients’ ability to consistently comply with data collection, thereby impacting the accuracy and reliability of health decision-making.

Overall, using DHT for the long-term monitoring of individuals with COPD offers more benefits than drawbacks. In the context of global digital transformation, medical digitalization is an inevitable trend. Therefore, limitations in equipment and urban-rural disparities will improve with advancements in information security and changes in insurance systems. Digitalization will significantly reduce overall health care costs, enhance precision, and transform personal health behaviors and motivations, ultimately achieving a patient-centered vision of health and well-being.

## Abbreviations

COPD: chronic obstructive pulmonary disease
DH: digital health
DHT: digital health technology
TA: thematic analysis

## Data Availability

The datasets generated during and/or analyzed during this study are not publicly available due to participant protection rights but are available from the corresponding author upon reasonable request.
